# Pretreatment with oral contraceptive pills in women with PCOS scheduled for IVF: a randomized clinical trial

**DOI:** 10.1093/hropen/hoae019

**Published:** 2024-04-04

**Authors:** Jun Gao, Qingyun Mai, Yiping Zhong, Benyu Miao, Minghui Chen, Lu Luo, Canquan Zhou, Ben W Mol, Xu Yanwen

**Affiliations:** Department of Obstetrics and Gynaecology, Reproductive Medicine Center, The First Affiliated Hospital, Sun Yat-Sen University, Guangzhou, China; Guangdong Provincial Key Laboratory of Reproductive Medicine, The First Affiliated Hospital, Sun Yat-Sen University, Guangzhou, China; Department of Obstetrics and Gynaecology, Guangdong Provincial Clinical Research Center for Obstetrical and Gynecological Diseases, The First Affiliated Hospital, Sun Yat-Sen University, Guangzhou, China; Department of Obstetrics and Gynaecology, Reproductive Medicine Center, The First Affiliated Hospital, Sun Yat-Sen University, Guangzhou, China; Guangdong Provincial Key Laboratory of Reproductive Medicine, The First Affiliated Hospital, Sun Yat-Sen University, Guangzhou, China; Department of Obstetrics and Gynaecology, Guangdong Provincial Clinical Research Center for Obstetrical and Gynecological Diseases, The First Affiliated Hospital, Sun Yat-Sen University, Guangzhou, China; Department of Obstetrics and Gynaecology, Reproductive Medicine Center, The First Affiliated Hospital, Sun Yat-Sen University, Guangzhou, China; Guangdong Provincial Key Laboratory of Reproductive Medicine, The First Affiliated Hospital, Sun Yat-Sen University, Guangzhou, China; Department of Obstetrics and Gynaecology, Guangdong Provincial Clinical Research Center for Obstetrical and Gynecological Diseases, The First Affiliated Hospital, Sun Yat-Sen University, Guangzhou, China; Department of Obstetrics and Gynaecology, Reproductive Medicine Center, The First Affiliated Hospital, Sun Yat-Sen University, Guangzhou, China; Guangdong Provincial Key Laboratory of Reproductive Medicine, The First Affiliated Hospital, Sun Yat-Sen University, Guangzhou, China; Department of Obstetrics and Gynaecology, Guangdong Provincial Clinical Research Center for Obstetrical and Gynecological Diseases, The First Affiliated Hospital, Sun Yat-Sen University, Guangzhou, China; Department of Obstetrics and Gynaecology, Reproductive Medicine Center, The First Affiliated Hospital, Sun Yat-Sen University, Guangzhou, China; Guangdong Provincial Key Laboratory of Reproductive Medicine, The First Affiliated Hospital, Sun Yat-Sen University, Guangzhou, China; Department of Obstetrics and Gynaecology, Guangdong Provincial Clinical Research Center for Obstetrical and Gynecological Diseases, The First Affiliated Hospital, Sun Yat-Sen University, Guangzhou, China; Department of Obstetrics and Gynaecology, Reproductive Medicine Center, The First Affiliated Hospital, Sun Yat-Sen University, Guangzhou, China; Guangdong Provincial Key Laboratory of Reproductive Medicine, The First Affiliated Hospital, Sun Yat-Sen University, Guangzhou, China; Department of Obstetrics and Gynaecology, Guangdong Provincial Clinical Research Center for Obstetrical and Gynecological Diseases, The First Affiliated Hospital, Sun Yat-Sen University, Guangzhou, China; Department of Obstetrics and Gynaecology, Reproductive Medicine Center, The First Affiliated Hospital, Sun Yat-Sen University, Guangzhou, China; Guangdong Provincial Key Laboratory of Reproductive Medicine, The First Affiliated Hospital, Sun Yat-Sen University, Guangzhou, China; Department of Obstetrics and Gynaecology, Guangdong Provincial Clinical Research Center for Obstetrical and Gynecological Diseases, The First Affiliated Hospital, Sun Yat-Sen University, Guangzhou, China; Department of Obstetrics and Gynaecology, The Ritchie Center, Monash University, Clayton, VIC, Australia; Aberdeen Centre for Women’s Health Research, Institute of Applied Health Sciences, School of Medicine, Medical Sciences and Nutrition, University of Aberdeen, Aberdeen, UK; Department of Obstetrics and Gynaecology, Reproductive Medicine Center, The First Affiliated Hospital, Sun Yat-Sen University, Guangzhou, China; Guangdong Provincial Key Laboratory of Reproductive Medicine, The First Affiliated Hospital, Sun Yat-Sen University, Guangzhou, China; Department of Obstetrics and Gynaecology, Guangdong Provincial Clinical Research Center for Obstetrical and Gynecological Diseases, The First Affiliated Hospital, Sun Yat-Sen University, Guangzhou, China

**Keywords:** PCOS, LH, GnRH antagonist, oral contraceptive pills, random-start ovarian stimulation

## Abstract

**STUDY QUESTION:**

What is the effect of pretreatment with oral contraceptive pills (OCPs) on oocyte and embryo quality and pregnancy rates in women with polycystic ovary syndrome (PCOS) scheduled for IVF/ICSI cycles?

**SUMMARY ANSWER:**

In women with PCOS who underwent a first or second IVF/ICSI cycle with a GnRH antagonist protocol and were randomized to start ovarian stimulation immediately, the quality of cleavage-stage embryos was non-inferior to pretreatment with OCP.

**WHAT IS KNOWN ALREADY:**

PCOS in Asian populations is characterized by high levels of circulating LH in the early follicular phase. Previous studies indicated that inappropriately high LH levels might affect oocyte maturation and fertilization rates, and impaired embryo quality, consequently resulting in higher rates of impaired pregnancy and miscarriage in women with PCOS. OCPs are frequently used as pretreatment to lower LH levels in PCOS patients.

**STUDY DESIGN, SIZE, DURATION:**

We performed a randomized controlled trial. After informed consent, women diagnosed with PCOS scheduled for their first or second IVF/ICSI cycle with a GnRH antagonist protocol were randomized to receive OCPs (OCP group) or start ovarian stimulation immediately, regardless of the day of the menstrual cycle (non-OCP group). Using a non-inferiority hypothesis, the sample size was calculated at 242 women. The study lasted from 7 February 2018 to 31 August 2021.

**PARTICIPANTS/MATERIALS, SETTING, METHODS:**

A total of 242 infertility patients with PCOS undergoing the first or second cycle of IVF or ICSI were enrolled and randomized into two groups. In the OCP group, recombinant FSH was started on Day 7 of the washout period after pretreatment with OCP. In the non-OCP group, recombinant FSH was started immediately regardless of the day of the menstrual cycle. All participants received standardized GnRH antagonist ovarian stimulation. The freeze-all strategy was applied to all participants. The primary outcome was the number of good-quality embryos on Day 3 after insemination. Secondary outcomes included the rates of blastocyst formation, implantation, clinical pregnancy, and live birth from the first frozen/warmed embryo transfer cycles and cumulative live birth rates.

**MAIN RESULTS AND THE ROLE OF CHANCE:**

We randomized 242 women to receive OCP (n = 121) or start immediately with ovarian stimulation (n = 121). The number of good-quality embryos on Day 3 in the OCP group was non-inferior to the non-OCP group (OCP group versus non-OCP group, 6.58 ± 4.93 versus 7.18 ± 4.39, AD −0.61, 95% CI: −1.86 to 0.65, *P *=* *0.34). The rates of blastocyst formation (55.4% versus 52.9%, relative risk (RR) 1.11, 95% CI: 0.96 to 1.28, *P *=* *0.17), implantation (63.0% versus 65.5%, RR 0.90, 95% CI: 0.53 to 1.53, *P *=* *0.79), clinical pregnancy (67.9% versus 68.8%, RR 0.96, 95% CI: 0.54 to 1.71, *P *=* *1.0), and live birth rate (52.8% versus 55.1%, RR 0.92, 95% CI: 0.53 to 1.56, *P *=* *0.79) of the first frozen/warmed embryo transfer cycles were all comparable between the OCP and non-OCP group, respectively. Cumulative live birth rates were also similar in the OCP and non-OCP groups (78.3% versus 83.5%, respectively RR 0.71, 95% CI: 0.36 to 1.42, *P *=* *0.39).

**LIMITATIONS, REASONS FOR CAUTION:**

Only patients with PCOS in Southern China were recruited. Therefore, caution is necessary when generalizing our results to all such patients with PCOS. Also, since a freeze-only strategy was used, the results of this study are only applicable when infertile women with PCOS undergo the freeze-only method. The obvious treatment difference between the two groups meant that the study was designed as an open-label study for women and doctors. The study had a randomized controlled design that minimized bias.

**WIDER IMPLICATIONS OF THE FINDINGS:**

Pretreatment with OCPs to lower LH levels in patients with PCOS before ovarian stimulation in IVF or ICSI cycles may not improve the quality of cleavage-stage embryos.

**STUDY FUNDING/COMPETING INTEREST(S):**

This study was funded by the National Key Research and Development Program of China (No. 2023YFC2705503). This study was supported in part by the Investigator-Initiated Studies Program (grant from MSD and Organon). BWM reports consultancy, travel support, and research funding from Merck. He reports consultancy from Organon and Norgine, and also reports holding stock from ObsEva. No conflicts of interest are declared for the other authors.

**TRIAL REGISTRATION NUMBER:**

Chinese Clinical Trial Registry (No. chiCTR1800014822). URL: https://www.chictr.org.cn/showproj.html?proj=25280

**TRIAL REGISTRATION DATE:**

7 February 2018.

**DATE OF FIRST PATIENT’S ENROLLMENT:**

22 February 2018.

WHAT DOES THIS MEAN FOR PATIENTS? Polycystic ovary syndrome (PCOS) is the most common cause of infertility caused by a lack of ovulation (release of an egg mid-cycle). A high level of circulating luteinizing hormone (LH) is an important feature of PCOS: LH normally stimulates ovulation and helps with the hormone production needed to support pregnancy. High LH levels, however, may be detrimental. This study was designed to explore whether, in IVF or ICSI cycles, pretreatment (before the standard ovarian stimulation) with oral contraceptive pills (OCPs) to lower LH levels in women with PCOS may improve the quality of embryos. Women were randomly assigned to one of two groups. One group was given OCP for 21 days before ovarian stimulation in the IVF or ICSI cycle and the other group was stimulated immediately, without OCP pretreatment. The results showed that the number of good-quality embryos and live birth rates were similar in the two groups, and therefore pretreatment with OCP in women with PCOS is not necessary.

## Introduction

Polycystic ovary syndrome (PCOS) is the most common cause of anovulatory infertility. A high level of circulating LH is an important biochemical feature of PCOS (Baldani *et al.*, 2012; Liu *et al.*, 2012). In women with PCOS, the prevalence of elevated LH varies between 35% and 75%, especially in Asian populations (Banaszewska *et al.*, 2003; Wang and Alvero, 2013; Endocrinology Subgroup and Expert Panel, Chinese Society of Obstetrics and Gyneocology, Chinese Medical Association, 2018). In a study by Chen *et al.* (2016) involving 1508 women with PCOS, the average ratio of LH to FSH was 1.5–1.6.

Previous studies indicated that inappropriately high LH levels might affect oocyte maturation and fertilization rates, and impaired embryo quality, consequently resulting in higher rates of impaired pregnancy and miscarriage in women with PCOS (Balen *et al.*, 1993a,b; Homburg *et al.*, 1993; Shoham *et al.*, 1993; Jabara and Coutifaris, 2003; Urman *et al.*, 2004; Santos et al., 2010). However, recent evidence from retrospective studies has shown that high LH levels had no obvious negative effect on embryo quality in women with PCOS (Chen *et al.*, 2016; Sun *et al.*, 2018; Singh *et al.*, 2021; Liu and Wang, 2023).

Clinically, there are several methods used to reduce LH levels in women with PCOS. Pituitary downregulation using GnRH agonists significantly lowers the LH level in women with PCOS (Homburg *et al.*, 1993; Balen *et al.*, 1993a). However, the GnRH agonist long protocol increases the risk of ovarian hyperstimulation syndrome (OHSS) in women with PCOS compared with the GnRH antagonist protocol. OCPs, widely used in women with PCOS with irregular menstrual cycles, can also suppress LH levels. OCP pretreatment before the GnRH antagonist protocol is therefore a reasonable choice to lower LH levels before ovarian stimulation.

The present study investigated whether not providing OCP to lower LH levels would affect oocyte/embryo quality in women with PCOS. We designed a single-center, open-label non-inferiority randomized controlled trial (RCT) to explore whether OCP pretreatment before the GnRH antagonist protocol in women with PCOS would improve embryo quality.

## Materials and methods

### Governance

We performed an RCT from 7 February 2018 to 31 August 2021, at the Reproductive Center of the First Affiliated Hospital of Sun Yat-sen University in Guangzhou, China. The study protocol was approved by the ethics committees for Clinical Research and Animal Trials of the First Affiliated Hospital of Sun Yat-sen University (No.: [2018]014). The study was conducted in accordance with the Declaration of Helsinki and good clinical practice guidelines. Our study was registered in the Chinese Clinical Trial Registry (registration number chiCTR1800014822) on 7 February 2018. All women provided written informed consent to participate in the study.

### Study population

Patients were eligible if they fulfilled the following inclusion criteria: diagnosis of PCOS according to the 2003 Rotterdam PCOS diagnostic criteria (Rotterdam ESHRE/ASRM-Sponsored PCOS Consensus Workshop Group, 2004); patient age 20–40 years; and scheduled for a first or second IVF/ICSI cycle. Indications for IVF included tubal pathology, male factor, combined tubal and male factors, or PCOS only. Women with endometriosis, endometrioma or submucous myoma, uterine malformations such as bicornuate uterus and uterine cavity adhesion, ovarian tumors, recurrent spontaneous abortion, and chromosomal abnormalities were not eligible.

### Randomization, blinding, and grouping

Women fulfilling the inclusion criteria were approached by their clinician and asked if they were willing to participate. Ultrasound was performed to confirm the absence of dominant follicle (follicles > 8 mm in size) development, and a urine pregnancy test was used to exclude pregnancy.

After obtaining written informed consent, eligible women were randomized using random numbers generated by SPSS software (version 24.0, IBM Corp., Armonk, NY, USA). Randomization was conducted by a research assistant of the hospital who was not involved in the recruitment and clinical management of the participants. The research assistant provided sealed envelopes with treatment allocation (the OCP group and the non-OCP group) to the study staff. The obvious treatment differences between the two groups meant that the study was designed as an open-label study for women and doctors. However, embryologists and IVF technicians who evaluated the oocytes and embryos were unaware of the treatment allocation.

Participants allocated to the OCP group were provided with OCPs containing ethinyl estradiol (0.03 mg) and drospirenone (3 mg). OCPs were administered daily for 21 days to induce menstruation, followed by 7 days of washout. Recombinant FSH (rFSH) was started on Day 7 of the washout period.

In participants allocated to the non-OCP group, rFSH was started immediately regardless of the day of the menstrual cycle. Otherwise, the groups were treated comparably.

### Controlled ovarian stimulation and oocyte retrieval

All participants underwent a fixed protocol of GnRH antagonist ovarian stimulation. First, an ultrasound was performed to confirm the absence of dominant follicles (follicle size > 8 mm). After confirmation, controlled ovarian stimulation (COS) was initiated using human rFSH (Puregon, Merck Sharp & Dohme, Ravensburg, Germany) daily for 3–4 days. The starting dose of rFSH was fixed according to the BMI of the patient (150 IU for BMI 19–25 kg/m^2^; 225 IU for BMI > 25.1 kg/m^2^). Transvaginal ultrasound was performed, and serum LH, estrogen, and progesterone levels were assessed to monitor follicle growth. The rFSH dose was adjusted according to the ovarian response. A GnRH antagonist (Ganirelix; Orgalutran, N. V. Organon, New Jersey City, NJ, USA) was administered s.c. at 0.25 mg daily on Day 5 or 6 of rFSH stimulation in the two groups. Triggering of the final oocyte maturation was performed when two follicles ≥18 mm or three follicles ≥17 mm were noted under ultrasound; a dual trigger [2000 IU hCG plus 0.2 mg of triptorelin (Decapeptyl, Ferring, Kiel, Germany)] or 250 μg of recombinant hCG [Ovidrel; Merck Serono, Modugno (Bari), Italy] was used. Blood samples were obtained 8–12 h after the dual trigger to determine LH, estradiol, and progesterone levels. If the LH level was <15 IU/l or the progesterone level was <3 ng/ml (Chang *et al.*, 2016), hCG 2000 IU was re-administered to the patient 14–16 h after the trigger to supplement the insufficient endogenous LH that could lead to reduced oocyte retrieval. Oocyte retrieval was performed 34–36 h after the trigger using the transvaginal ultrasound-guided puncture of follicles.

### IVF and embryo culture

Standard laboratory protocols for conventional IVF and ICSI (Magli *et al.*, 2008) were performed. ICSI was only performed for male factor infertility, according to ESHRE guidelines (ESHRE Capri Workshop Group, 2007). Evaluation of embryo quality on Day 3 was performed according to the modified Istanbul consensus (Alpha Scientists in Reproductive Medicine and ESHRE Special Interest Group of Embryology, 2011). Good-quality embryos were defined as 7–9 cells with 0–20% cytoplasmic fragments and an even size on the third day after fertilization. The blastocyst score on Day 5 or 6 was assessed according to the Gardner morphological criteria (Gardner *et al.*, 2000), based on the degree of expansion and the development of the inner cell mass and trophectoderm. A freeze-all strategy was applied for all participants.

### Embryo transfer and luteal-phase support

A hormone replacement therapy-based protocol was performed for endometrial preparation in the frozen/warmed embryo transfer (FET) cycle. On Days 2–3 of the second menstrual cycle (either spontaneous or induced by progesterone) after oocyte retrieval, women received oral estradiol valerate (Progynova, Delpharm Lille, Lys-Lez-Lannoy, France) for endometrial preparation. Intramuscular progesterone (40–60 mg) or vaginal progesterone gel (Crinone, Merck Serono, SP, Brazil; 90 mg/day) with oral dydrogesterone (10 mg twice daily) was administered when endometrial thickness reached 8 mm, and estradiol valerate was used for ≥10 days. Embryos were transferred on the fourth (cleavage-stage embryo) or sixth (blastocyst) day after progesterone initiation. Twelve days after the embryo transfer, serum β-hCG was measured. If biochemical pregnancy was achieved, clinical pregnancy was confirmed on the basis of the detection of gestational sacs by ultrasonography at 4–5 weeks after the embryo transfer. The luteal-phase support continued until 10 weeks after conception. All pregnancy and neonatal outcomes were obtained through review of medical records.

### Serum FSH, LH, estradiol, and progesterone assessment

Basal FSH, LH, and estradiol levels were determined in the morning of Days 2–5 of the menstrual cycle (either spontaneous or induced by progesterone). Serum FSH, LH, estradiol, and progesterone levels were determined using an automated Elecsys immunoanalyzer (Abbott Ireland Diagnostics Division, Longford, Ireland). Intra- and inter-assay coefficients of variation were <4% and <5%, respectively, for LH; <5% and <6%, respectively, for FSH; <7% and <8%, respectively, for estradiol; and <6% and <7%, respectively, for progesterone. The assays were carried out in batches.

### Outcome measures

The primary outcome of the study was the number of good-quality embryos on Day 3 after insemination. Secondary outcomes included the rates of blastocyst formation, implantation, clinical pregnancy, live birth pregnancy during the first FET cycle, and cumulative live births rates and neonatal gestational age, birthweight, and fetal gender.

The blastocyst formation rate was defined as the ratio of the number of blastocysts to the number of Day 3 embryos cultured for blastocyst formation. Clinical pregnancy was defined as the presence of an intrauterine gestation sac at 6–7 weeks of gestation. Live birth was defined as delivery of any viable infant at 28 weeks or more of gestation. Cumulative live births were defined as live birth resulting from pregnancies that occur within 24 months from the day of ovarian stimulation. The safety outcomes included moderate to severe OHSS (Practice Committee of the American Society for Reproductive Medicine, 2016), miscarriage, and ectopic pregnancy.

### Sample size

The sample size was calculated using PASS15 software (NCSS, LLC, East Kaysville, UT, USA). We hypothesized that immediate start with ovarian stimulation was non-inferior as compared to pretreatment with OCP. Non-inferiority was assumed to be proven if the lower boundary of the two-sided 95% CI did not exceed −2.0 good-quality embryos on Day 3. Assuming that the actual distribution was normal and the SD was 5.0, a total of 210 participants (in a 1:1 ratio) were required to achieve 80% power to detect non-inferiority with a one-sided significance level of 0.025. The margin of non-inferiority was −2.0, and the true difference between the means was assumed to be 0. Considering a 15% dropout rate in the study, we planned to include two groups of 121 women (242 women).

### Statistical analysis

All randomized participants were included in the intent-to-treat analysis. Values are reported as the median (interquartile range) for non-normally distributed variables, the mean ± SD for normally distributed variables, or as numbers (percentages) for categorical variables. We compared baseline data between the two groups. After confirming that the baseline data between the two groups were balanced and comparable, the outcomes of COS and pregnancy outcome were compared. The differences in continuous variables between the intervention and control groups were analyzed using the independent sample Student’s *t-*test if the data followed normal distributions; otherwise, Mann–Whitney *U* tests were applied. Categorical variables were analyzed using the chi-squared test. SPSS version 24.0 (IBM, Armonk, NY, USA) was used for the data analysis. A *P*-value of <0.05 indicated statistical significance.

## Results

### Study participants

**Figure 1.**
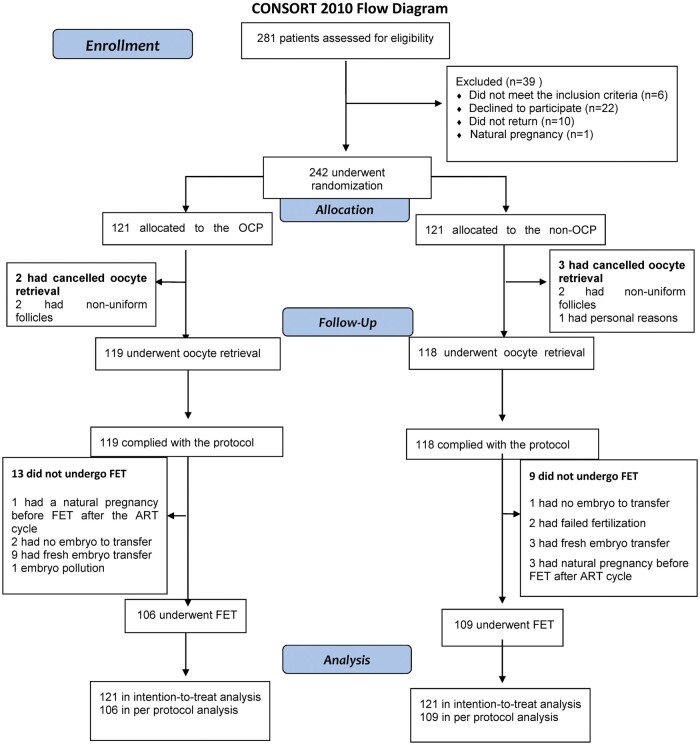
**Flowchart of patient enrollment, allocation, follow-up, and analysis in a randomized clinical trial of pretreatment with oral contraceptive pills in women with PCOS undergoing IVF**. OCP, oral contraceptive pills; FET, frozen/warmed embryo transfer.

From February 2018 to August 2021, 281 infertile women with PCOS undergoing their first or second IVF cycle were enrolled. There were 39 participants excluded from the study for various reasons (Fig. 1). A total of 242 participating participants were randomized to the OCP (n = 121) or non-OCP (n = 121) group.

The baseline characteristics of the participants were comparable between the two groups (Table 1). Hormonal and metabolic indices were similar between the two groups (Supplementary Table S1).

**Table 1.** Baseline characteristics of patients in the OCP and non-OCP groups.

	OCP group (n = 121)	Non-OCP group (n = 121)
Age (years)	29 (21, 39)	29 (21, 37)
BMI (kg/m^2^)	22.48 ± 2.93	22.67 ± 3.29
Basal FSH (U/l)	4.96 ± 1.10	5.04 ± 1.09
Basal LH (U/l)	7.32 ± 4.29	7.51 ± 4.28
Ratio of LH/FSH	1.47 ± 0.77	1.50 ± 0.83
Testosterone (ng/ml)	0.50 ± 0.55	0.44 ± 0.13
No. of IVF cycles	1.06 ± 0.23	1.05 ± 0.22
Duration of attempts to conceive (years)	3.62 ± 2.40	3.27 ± 1.82
Previous conception	46 (38.02%)	39 (32.23%)
Main causes of infertility		
Tubal factors	57 (47.11%)	49 (40.50%)
Male factors	29 (23.97%)	32 (26.45%)
Type of insemination		
IVF	89 (73.55%)	84 (69.42%)
ICSI	29 (23.97%)	31 (25.62%)
Half-ICSI	3 (2.48%)	6 (5.0%)

Age is presented as median (minimum, maximum). Other values are presented as mean ± SD or n (%). OCP, oral contraceptive pill; half-ICSI, in cases with the risk of fertilization failure, half oocytes were inseminated by conventional IVF and half were inseminated by ICSI. The baseline characteristics of the participants were comparable between the two groups.

### Outcomes of COS and FET

The mean cycle day of gonadotrophin initiation was significantly lower in the OCP group than in the non-OCP group, mainly because of the random start protocol in the non-OCP group. As expected, the OCP group had lower LH levels than the non-OCP group on the day of gonadotrophin initiation (Table 2). The LH/FSH ratio on the day of gonadotrophin initiation was also lower in the OCP group. The OCP group required significantly less gonadotrophin and a shorter duration of stimulation (Table 2). Two of 101 women in the OCP group and 2 of 109 women in the non-OCP group had hCG 2000 IU administered 14–16 h after the dual trigger because of inadequate endogenous LH levels. The number of retrieved oocytes was significantly lower in the OCP group than in the non-OCP group. The metaphase II oocyte rate, the fertilization rate, and two pronuclei (2PN) rate in the ICSI cycle were similar between the two groups. However, the fertilization and 2PN rates in the IVF cycles were significantly higher in the OCP group than in the non-OCP group (Table 2).

**Table 2.** Outcomes of controlled ovarian hyperstimulation in the OCP versus non-OCP group.

	OCP group		Non-OCP group		Absolute difference between groups (95% CI)	*P*-value
	N	Value	N	Value		
Starting dose of rFSH (IU)	119	161.55 ± 30.15	120	162.9 ± 29.70	−1.36 (−18.99, 6.26)	0.725
Menstrual cycle day on the day of initiation	121	4 (3, 4)	121	6 (3, 22)	—	<0.001
FSH on the day of Gn initiation (U/l)^a^	120	5.05 ± 1.17	115	5.00 ± 0.99	0.05 (−0.23, 0.33)	0.716
LH on the day of Gn initiation (U/l)^a^	120	5.14 (3.55, 6.74)	116	7.60 (4.24, 11.63)	—	<0.001
LH/FSH ratio on the day of Gn initiation^a^	120	1.01 (0.76, 1.33)	115	1.53 (0.83, 2.26)	—	<0.001
Estradiol level on day of Gn initiation (pg/ml)^a^	120	38.72 ± 17.55	116	35.42 ± 14.82	3.29 (−0.88, 7.47)	0.121
LH on D5 of Gn initiation (U/l)	119	2.85 (1.94, 5.02)	120	5.37 (2.51, 15.11)	—	<0.001
No. of days of ovarian stimulation	119	7.98 ± 1.19	118	8.40 ± 1.16	−0.42 (−0.72, −0.11)	0.007
Total Gn dose (U)	119	1 215.23 ± 304.0	118	1 311.02 ± 364.20	−95.79 (−181.61, −9.96)	0.029
LH level on hCG trigger day (U/l)	119	1.96 (1.28, 3.36)	118	3.22 (1.69, 4.90)	—	0.001
Estradiol level on hCG trigger day (pg/ml)	119	4 213.28 ± 1022.15	118	4 220.55 ± 1145.14	−7.27 (−285.01, 270.46)	0.959
Progesterone level on hCG trigger day (ng/ml)	119	0.95 ± 0.47	118	1.05 ± 0.71	−0.10 (−0.25, 0.05)	0.201
Trigger	119		118			
Dual trigger		101 (84.87%)		109 (92.37%)	0.46 (0.20, 1.08)	0.101
rhCG trigger		16 (13.45%)		8 (6.78%)	2.14 (0.88, 5.20)	0.130
GnRH-a trigger		2 (1.68%)		1 (0.85%)	2.0 (0.18, 22.36)	1.0
No. of oocytes retrieved	119	22.82 ± 11.56	118	26.63 ± 11.10	−3.81 (−6.71, −0.91)	0.010
MII oocyte rate in ICSI cycle (%)	30	82.58 ± 13.90	35	82.08 ± 15.58	0.5 (−6.87, 7.88)	0.892
Fertilization rate in ICSI cycle (%)	30	82.39 ± 11.31	35	81.12 ± 17.36	1.27 (−6.13, 8.67)	0.732
2PN rate in ICSI cycles (%)	30	77.21 ± 14.05	35	78.77 ± 17.39	−1.56 (−9.49, 6.36)	0.695
Fertilization rate in IVF cycles (%)	90	84.99 ± 15.49	89	76.75 ± 19.37	8.24 (3.07, 13.41)	0.002
2PN rate in IVF cycles (%)	90	70.62 ± 17.86	89	61.94 ± 19.27	8.68 (3.20, 14.16)	0.002
Cleavage rate (%)	119	95.91 ± 8.10	118	94.72 ± 10.71	1.19 (−1.24, 3.61)	0.337

Values are presented as mean ± SD, median (interquartile range), or n (%).

a In the OCP group, one patient did not have a blood sample for the detection of FSH, LH, and estradiol. In the non-OCP group, six patients did not have blood samples for the detection of FSH, five patients did not have blood samples for the detection of LH and estradiol. In the OCP group, three patients with half-ICSI, and in the non-OCP group six patients with half-ICSI were analyzed separately in the IVF cycle and ICSI cycle. rFSH, recombinant FSH; Gn, gonadotrophin; GnRH-a, GnRH agonist; MII, metaphase II; 2PN, double pronuclear; OCP, oral contraceptive pill.

Thirteen participants in the OCP group and nine participants in the non-OCP group did not undergo FET (Fig. 1). The FET outcomes of 106 participants in the OCP group and 109 participants in the non-OCP group were analyzed. One patient in each group was lost to follow-up after 12 weeks of gestation. There were no significant differences between the groups in terms of the FET endometrial preparation protocol, endometrial thickness, type of transferred embryos, or number of embryos transferred (Supplementary Table S2).

### Primary endpoint and pregnancy outcomes

The number of good-quality embryos on Day 3 in the non-OCP group was not inferior to that in the OCP group (6.96 ± 4.51 versus 6.25 ± 4.88, respectively, absolute difference (AD) 0.71, 95% CI: −0.50 to 1.9), *P *=* *0.25 for the intention to treat (ITT) population, while in the per-protocol (PP) analysis, the number of good-quality embryos on Day 3 was 7.18 ± 4.39, versus 6.58 ± 4.93 (AD 0.61, 95% CI: −0.65 to 1.86, *P *=* *0.34, respectively). There were no significant differences between the two groups in the rate of blastocyst formation. Incidence of moderate or severe OHSS was 6.61% in the OCP group and 10.74% in the non-OCP group (relative risk 0.59, 95% CI: 0.24 to 1.48, *P* = 0.36). The results were similar for the ITT and PP analyses (Table 3).

**Table 3.** Primary, secondary, and safety endpoints for intention-to-treat analysis and per-protocol analysis.

Intention-to-treat analysis					
	OCP group	Non-OCP group	Absolute difference between groups (95% CI)	Rate ratio (95% CI)	*P*-value
	(n = 121)	(n = 121)			
No. of good-quality embryos on Day 3	6.25 ± 4.88	6.96 ± 4.51	−0.71 (−1.91, 0.50)	—	0.249
Blastocyst formation rate (%)	778/1445 (53.84%)	877/1653 (53.06%)	—	1.03 (0.90, 1.19)	0.665
Incidence of moderate or severe ovarian hyperstimulation syndrome (%)	8 (6.61%)	13 (10.74%)	—	0.59 (0.24, 1.48)	0.361
Cumulative live birth rate（%）^a^	90 (74.4%)	94 (77.7%）	—	0.83 (0.46, 1.51)	0.652

Per-protocol analysis					
	OCP group	Non-OCP group	Absolute difference between groups (95% CI)	Rate ratio (95% CI)	*P*-value
	(n = 106)	(n = 109)			
No. of good-quality embryos on Day 3	6.58 ± 4.93	7.18 ± 4.39	−0.61（−1.86, 0.65）	—	0.34
Blastocyst formation rate (%)	751/1356 (55.38%)	843/1595 (52.85%)	—	1.11 (0.96, 1.28)	0.17
Implantation rate (%)	75/119 (63.03%)	76/116 (65.52%)	—	0.90 (0.53, 1.53)	0.786
Clinical pregnancy rate (%)	72 (67.92%)	75 (68.81%)	—	0.96 (0.54, 1.71)	1.0
Live birth rate (%)^a^	56 (52.83%)	60 (55.05%)	—	0.92 (0.53, 1.56)	0.785
Cumulative live birth rate（%）^a^	83 (78.3%)	91 (83.5%)		0.71 (0.36, 1.42)	0.387
First-trimester miscarriage rate (%)	13 (18.06%)	11 (14.67%)	—	1.25 (0.53, 2.92)	0.658
Incidence of moderate or severe ovarian hyperstimulation syndrome (%)	6 (5.66%)	12 (11.01%)	—	0.54 (0.19, 1.50)	0.31
Ectopic pregnancy (%)	0 (0.0)	0 (0.0)	—	—	—

Unless otherwise stated, values are presented as mean ± SD or n (%).

a One patient in each group was lost to follow-up after 12 weeks of gestation.

OCP, oral contraceptive pill.

The clinical outcomes after the first FET cycle, including the implantation rate, clinical pregnancy rate, live birth pregnancy rate, and first trimester miscarriage rate, were similar between the two groups (Table 3). There were no ectopic pregnancies in either group. Cumulative live births were also similar between the two groups (Table 3). Neonatal gestational age, birthweight and fetal gender were also similar between the two groups (Table 4).

**Table 4.** Newborns’ gestational age, birthweight, and male gender in the OCP and non-OCP groups.

	OCP group	Non-OCP group	Absolute difference between groups (95% CI)	Rate ratio (95% CI)	*P*-value
	(n = 56)	(n = 60)			
Gestational age at delivery（days）	272.16 ± 12.4	270.35 ± 15.19	1.81（−3.32, 6.94）	—	0.486
Single birth (n)	52	58		—	
Single birthweight (g)	3 286.54 ± 374.6	3 216.21 ± 510.5	70.33（−100.58, 241.24）	—	0.416
Twins birth (n)	4	2	—	—	
Twins’ birthweight (g)	2 418.75 ± 225.10	2 047.50 ± 520.09	371.25（−94.70, 837.20）	—	0.106
Male gender	33 (55%)	25 (40.3%)	—	1.81（0.88, 3.71）	0.147

Unless otherwise stated, values are presented as mean ± SD or n (%).

## Discussion

In this RCT study, we confirmed that in women with PCOS scheduled for IVF, no pretreatment was non-inferior to pretreatment with OCP. Apparently, high LH levels on the day of gonadotrophin initiation did not have an obvious negative impact on embryo quality. Lowering the LH level with OCP before ovarian stimulation might be unnecessary.

PCOS frequently presents with abnormally high basal LH levels, especially in Asian populations. The impact of high basal LH levels on reproductive outcomes remains unclear. Previous studies suggested that high basal LH levels have a deleterious effect on embryo quality and implantation rates, and may be a causal factor in early pregnancy loss (Homburg *et al.*, 1988; Tesarik and Mendoza, 2002). However, others came to different conclusions. Studies showed that suppressing LH levels using GnRH analogs did not decrease the miscarriage rate in women with PCOS (Clifford *et al.*, 1996; Ludwig *et al.*, 1999). Recent studies showed that a high basal LH level and/or a high LH/FSH ratio in women with PCOS had no significant impact on the number of top-quality embryos, the clinical pregnancy rate (Sun *et al.*, 2018; Singh *et al.*, 2021; Fu and Kuang, 2023; Liu and Wang, 2023), or even the miscarriage rate (Sun *et al.*, 2018; Fu and Kuang, 2023). However, all the above studies were retrospective analyses. Prospective RCT studies are lacking on this issue, begging the question: is it necessary to lower LH levels in women with PCOS before initiating the GnRH antagonist protocol?

Oral contraceptives are commonly used to schedule the day of initiation in an IVF cycle and can lower the LH level. Clear consensus is lacking on whether pretreatment with combined oral contraceptives in COS protocols has a negative effect on pregnancy outcome. To control the possible adverse impact of oral contraceptives on oocyte quality, we initiated COS after 7 days of washout in our study. As expected, the LH level on the day of gonadotrophin initiation was significantly lower in the OCP group than in the non-OCP group, although the levels were comparable before OCP intervention.

Our study aimed to determine whether lowering LH levels would benefit oocyte quality; therefore, we set the number of good-quality cleavage-stage embryos as our primary endpoint. The results showed that the number of Day 3 good-quality embryos was similar between the two groups. In the first FET cycle after COS, there were no significant differences between the two groups in terms of the implantation, clinical pregnancy, miscarriage, and live birth rates. Our study is in line with previous retrospective studies, which showed that elevated LH levels in women with PCOS undergoing IVF did not have a negative effect on oocyte and embryo quality (Sun *et al.*, 2018; Singh *et al.*, 2021; Fu and Kuang, 2023; Liu and Wang, 2023) or pregnancy outcomes after FET (Sun *et al.*, 2018). In addition, our results showed that cumulative live birth rates did not differ between the two groups. Our results implied that lowering the LH level before COS in women with PCOS might be unnecessary.

We did not expect that the duration of ovarian stimulation would be significantly shorter in the OCP group than in the non-OCP group. In women with a normal response, oral contraceptives prolong the duration of stimulation and increase the total consumption of gonadotrophin (Rombauts *et al.*, 2006). The discrepancy between women with a normal response and our women with PCOS might reflect the degree of LH level reduction after OCP intervention. Rombauts *et al.* (2006) reported that oral contraceptive-scheduled group stimulation with rFSH commenced 2 days after OCP was discontinued; however, in our study, gonadotrophin stimulation was initiated 7 days after OCP discontinuation. More importantly, the serum LH level was 0.9 IU/l in the Rombauts *et al.* (2006) study, indicating a more profound pituitary suppression than that noted in our study (mean LH level = 5.32 IU/l). In other words, the relatively normal LH levels in the OCP group in our study might not have prolonged the duration of COS.

We found that the fertilization rate in the IVF cycles was significantly lower in the non-OCP group. Singh *et al.* (2021) also observed that the fertilization rate was lower in the group with higher basal LH levels, although the reason for this was unclear. We speculated that women with high LH levels might have smaller follicles after ovulation induction, resulting in a lower fertilization rate. However, nearly 70% of our women underwent IVF; therefore, it was impossible to compare the actual oocyte maturation rate between the two groups. This interesting phenomenon will be the subject of our future research.

Although we used the freeze-all strategy, a moderate/severe OHSS rate was still notable in both groups, especially in the non-OCP group (10.74%). This might have occurred for several reasons. First, we used a fixed initial dose according to patient BMI. The FSH doses were adjusted according to the ovarian response after 3–4 days. Although women with PCOS usually have a higher BMI, the initial dose might still be too high because of ample ovarian antral follicles. Second, the numbers of retrieved oocytes in both groups were high in our study. Finally, most women in our study were triggered with a short-acting GnRH agonist accompanied with small-dose hCG (2000 IU) in case of inadequate endogenous LH surge after GnRH agonist administration, and the latter might have increased the risk of OHSS.

To the best of our knowledge, this is the first RCT to focus on the possible benefits of lowering high LH levels in women with PCOS. Our study provides solid evidence that not suppressing LH was non-inferior to pretreatment with OCP. In addition, pretreatment with OCP may increase the risk of thrombo-embolic events in PCOS patients who will end up with grossly elevated serum levels of estradiol after COS. Furthermore, our control group was designed to have a random start (median 6 days of menstruation) unlike the traditional initiation time. Our study showed that withdrawal bleeding before COS was unnecessary for women with PCOS if the freeze-all strategy was used.

There are several limitations to our study. Only women with PCOS in Southern China were recruited; therefore, caution should be exercised when generalizing our results to all women with PCOS. Also, since a freeze-only strategy was used, the results of this study only are mainly applicable when infertile women with PCOS undergo the freeze-only method. Furthermore, because of the obvious treatment difference between the two groups, the study was designed as an open-label study for women and doctors. However, the study had a randomized controlled design that minimized bias.

## Conclusion

Our study showed that not suppressing LH was non-inferior to pretreatment with OCP before IVF in the GnRH antagonist protocol.

## Supplementary Material

hoae019_Supplementary_Data

## Data Availability

Individual deidentified participant data that underlie the results reported in this article (including text, tables, figures, and appendices) will be shared. Study protocol will be shared. Data will be available beginning 6 months and ending 5 years following article publication. Investigators whose proposed use of the data has been approved by an independent review committee identified for this purpose will be approved. Proposals should be directed to xuyanwen@mail.sysu.edu.cn. Data are available for 5 years at a third-party website (http://www.medresman.org.cn/uc/projectsh/projectedit.aspx?proj=4866).

## References

[hoae019-B1] Alpha Scientists in Reproductive Medicine and ESHRE Special Interest Group of Embryology. The Istanbul consensus workshop on embryo assessment: proceedings of an expert meeting. Hum Reprod 2011;26:1270–1283. 21502182 10.1093/humrep/der037

[hoae019-B2] Baldani DP , Skrgatić L, Goldstajn MS, Zlopasa G, Oguić SK, Canić T, Piljek AN. Clinical and biochemical characteristics of polycystic ovary syndrome in Croatian population. Coll Antropol 2012;36:1413–1418. 23390843

[hoae019-B3] Balen AH , Tan SL, Jacobs HS. Hypersecretion of luteinising hormone: a significant cause of infertility and miscarriage. Br J Obstet Gynaecol1993a;100:1082–1089. 8297840 10.1111/j.1471-0528.1993.tb15170.x

[hoae019-B4] Balen AH , Tan SL, MacDougall J, Jacobs HS. Miscarriage rates following in-vitro fertilization are increased in women with polycystic ovaries and reduced by pituitary desensitization with buserelin. Hum Reprod1993b;8:959–964. 8345091 10.1093/oxfordjournals.humrep.a138174

[hoae019-B5] Banaszewska B , Spaczyński RZ, Pelesz M, Pawelczyk L. Incidence of elevated LH/FSH ratio in polycystic ovary syndrome women with normo- and hyperinsulinemia. Rocz Akad Med Bialymst 2003;48:131–134. 14737959

[hoae019-B6] Chang FE , Beall SA, Cox JM, Richter KS, DeCherney AH, Levy MJ. Assessing the adequacy of gonadotropin-releasing hormone agonist leuprolide to trigger oocyte maturation and management of inadequate response. Fertil Steril 2016;106:1093–1100.e3. 27341988 10.1016/j.fertnstert.2016.06.013

[hoae019-B7] Chen ZJ , Shi Y, Sun Y, Zhang B, Liang X, Cao Y, Yang J, Liu J, Wei D, Weng N et al Fresh versus frozen embryos for infertility in the polycystic ovary syndrome. N Engl J Med 2016;375:523–533. 27509101 10.1056/NEJMoa1513873

[hoae019-B8] Clifford K , Rai R, Watson H, Franks S, Regan L. Does suppressing luteinising hormone secretion reduce the miscarriage rate? Results of a randomised controlled trial. BMJ 1996;312:1508–1511. 8646142 10.1136/bmj.312.7045.1508 PMC2351255

[hoae019-B9] Endocrinology Subgroup and Expert Panel, Chinese Society of Obstetrics and Gyneocology, Chinese Medical Association. [Chinese guideline for diagnosis and management of polycystic ovary syndrome]. Zhonghua Fu Chan Ke Za Zhi 2018;53:2–6 (in Chinese). 29374878 10.3760/cma.j.issn.0529-567X.2018.01.002

[hoae019-B10] ESHRE Capri Workshop Group. Intracytoplasmic sperm injection (ICSI) in 2006: evidence and evolution. Hum Reprod Update 2007;13:515–526. 17630396 10.1093/humupd/dmm024

[hoae019-B11] Fu W , Kuang Y. Role of luteinizing hormone elevation in outcomes of ovulation induction with letrozole for polycystic ovary syndrome. Front Med (Lausanne) 2023;10:1113840. 37144035 10.3389/fmed.2023.1113840 PMC10151707

[hoae019-B12] Gardner DK , Lane M, Stevens J, Schlenker T, Schoolcraft WB. Blastocyst score affects implantation and pregnancy outcome: towards a single blastocyst transfer. Fertil Steril 2000;73:1155–1158. 10856474 10.1016/s0015-0282(00)00518-5

[hoae019-B13] Homburg R , Armar NA, Eshel A, Adams J, Jacobs HS. Influence of serum luteinising hormone concentrations on ovulation, conception, and early pregnancy loss in polycystic ovary syndrome. BMJ 1988;297:1024–1026. 3142595 10.1136/bmj.297.6655.1024 PMC1834779

[hoae019-B14] Homburg R , Levy T, Berkovitz D, Farchi J, Feldberg D, Ashkenazi J, Ben-Rafael Z. Gonadotropin-releasing hormone agonist reduces the miscarriage rate for pregnancies achieved in women with polycystic ovarian syndrome. Fertil Steril 1993;59:527–531. 8458452 10.1016/s0015-0282(16)55794-x

[hoae019-B15] Jabara S , Coutifaris C. In vitro fertilization in the PCOS patient: clinical considerations. Semin Reprod Med 2003;21:317–324. 14593555 10.1055/s-2003-43310

[hoae019-B16] Liu N , Ma Y, Wang S, Zhang X, Zhang Q, Zhang X, Fu L, Qiao J. Association of the genetic variants of luteinizing hormone, luteinizing hormone receptor and polycystic ovary syndrome. Reprod Biol Endocrinol 2012;10:36. 22546001 10.1186/1477-7827-10-36 PMC3403896

[hoae019-B17] Liu Z , Wang KH. Effect of basal luteinizing hormone (bLH) level on in vitro fertilization/intra-cytoplasmic injections (IVF/ICSI) outcomes in polycystic ovarian syndrome (PCOS) patients. BMC Pregnancy Childbirth 2023;23:618. 37644399 10.1186/s12884-023-05944-4 PMC10466855

[hoae019-B18] Ludwig M , Finas D, Al-Hasani S, Diedrich K, Ortmann O. Oocyte quality and treatment outcome in intracytoplasmic sperm injection cycles of polycystic ovarian syndrome patients. Hum Reprod 1999;14:354–358. 10099978 10.1093/humrep/14.2.354

[hoae019-B19] Magli MC , Van den Abbeel E, Lundin K, Royere D, Van der Elst J, Gianaroli L; Committee of the Special Interest Group on Embryology. Revised guidelines for good practice in IVF laboratories. Hum Reprod 2008;23:1253–1262. 18375408 10.1093/humrep/den068

[hoae019-B20] Practice Committee of the American Society for Reproductive Medicine. Prevention and treatment of moderate and severe ovarian hyperstimulation syndrome: a guideline. Fertil Steril 2016;106:1634–1647. 27678032 10.1016/j.fertnstert.2016.08.048

[hoae019-B21] Rombauts L , Healy D, Norman RJ; Orgalutran Scheduling Study Group. A comparative randomized trial to assess the impact of oral contraceptive pretreatment on follicular growth and hormone profiles in GnRH antagonist-treated patients. Hum Reprod 2006;21:95–103. 16253978 10.1093/humrep/dei302

[hoae019-B22] Rotterdam ESHRE/ASRM-Sponsored PCOS Consensus Workshop Group. Revised 2003 consensus on diagnostic criteria and long-term health risks related to polycystic ovary syndrome. Fertil Steril 2004;81:19–25. 10.1016/j.fertnstert.2003.10.004 14711538

[hoae019-B23] Santos M , Kuijk E, Macklon N. The impact of ovarian stimulation for IVF on the developing embryo. Reproduction 2010;139:23–34. 19710204 10.1530/REP-09-0187

[hoae019-B24] Shoham Z , Jacobs HS, Insler V. Luteinizing hormone: its role, mechanism of action, and detrimental effects when hypersecreted during the follicular phase. Fertil Steril 1993;59:1153–1161. 8495757 10.1016/s0015-0282(16)55968-8

[hoae019-B25] Singh N , Mishra N, Dogra Y. Do basal luteinizing hormone and luteinizing hormone/follicle-stimulating hormone ratio have significance in prognosticating the outcome of in vitro fertilization cycles in polycystic ovary syndrome? J Hum Reprod Sci 2021;14:21–27. 34083988 10.4103/jhrs.JHRS_96_20 PMC8057154

[hoae019-B26] Sun L , Ye J, Wang Y, Chen Q, Cai R, Fu Y, Tian H, Lyu Q, Lu X, Kuang Y. Elevated basal luteinizing hormone does not impair the outcome of human menopausal gonadotropin and medroxyprogesterone acetate treatment cycles. Sci Rep 2018;8:13835. 30217999 10.1038/s41598-018-32128-4 PMC6138741

[hoae019-B27] Tesarik J , Mendoza C. Effects of exogenous LH administration during ovarian stimulation of pituitary down-regulated young oocyte donors on oocyte yield and developmental competence. Hum Reprod 2002;17:3129–3137. 12456612 10.1093/humrep/17.12.3129

[hoae019-B28] Urman B , Tiras B, Yakin K. Assisted reproduction in the treatment of polycystic ovarian syndrome. Reprod Biomed Online 2004;8:419–430. 15149566 10.1016/s1472-6483(10)60926-1

[hoae019-B29] Wang S , Alvero R. Racial and ethnic differences in physiology and clinical symptoms of polycystic ovary syndrome. Semin Reprod Med 2013;31:365–369. 23934697 10.1055/s-0033-1348895

