# Supplementary material for: Pretreatment with oral contraceptive pills in women with PCOS scheduled for IVF: a randomized clinical trial
Source: Hum Reprod Open. 2024 Apr 4;2024(2):hoae019. doi: 10.1093/hropen/hoae019 (PMC11653855; doi:10.1093/hropen/hoae019)
Supplement: hoae019_Supplementary_Data [file hoae019_supplementary_data.docx]

**Supplementary Table S1.** Hormonal and metabolic indices of the study population in OCP group and non-OCP group.

|  | OCP Group (n = 121) | | Non-OCP Group (n = 121) | | *P-* value |
| --- | --- | --- | --- | --- | --- |
|  | No. of patients | value | No. of patients | value |  |
| DHEA (μmol/L) | 89 | 8.28 ± 3.42 | 98 | 8.72 ± 3.13 | 0.366 |
| AMH (ng/mL) | 90 | 10.56 ± 6.20 | 86 | 10.54 ± 5.58 | 0.987 |
| Triglyceride (mmol/L) | 110 | 1.37 ± 0.75 | 107 | 1.31 ± 0.77 | 0.523 |
| Cholesterol (mmol/L) | 110 | 5.06 ± 0.95 | 107 | 5.06 ± 0.87 | 0.980 |
| High‑density lipoprotein (mmol/L) | 108 | 1.37 ± 0.31 | 106 | 1.37 ± 0.31 | 0.967 |
| Low-density lipoprotein (mmol/L) | 109 | 3.15 ± 0.69 | 106 | 3.19 ± 0.66 | 0.677 |
| Fasting blood glucose (mmol/L) | 115 | 4.87 ± 0.55 | 119 | 4.74 ± 0.75 | 0.135 |
| Fasting insulin (μU/mL) ^a^ | 102 | 9.34 (5.93, 13.79) | 108 | 8.37 (6.09, 12.52) | 0.260 |
| HOMA-IR ^a^ | 102 | 1.96 (1.25, 3.18) | 108 | 1.77 (1.32, 2.59) | 0.188 |

Values are presented as the mean ± SD, or the median (interquartile range). The variables marked with an a were non-normally distributed variables and used Mann–Whitney U tests; the unlabeled variables obeyed a normal distribution, and the Student’s t-test was used. OCP, Oral contraceptive pill; DHEA, dehydroepiandrosterone; AMH, anti-Mullerian hormone; HOMA-IR = (fasting blood glucose × fasting insulin) /22.5.

**Supplementary Table S2** FET cycle characteristics in OCP group and non-OCP group.

|  | | OCP Group (n = 106) | Non-OCP Group (n = 109) | *P*-value |
| --- | --- | --- | --- | --- |
| Endometrial preparation protocol | |  |  |  |
| HRT | | 94 (88.68%) | 92 (84.40 %) | 0.426 |
|  | LPS with Intramuscular progesterone | 36(38.30 %) | 48(52.17 %) | 0.077 |
|  | LPS with vaginal progesterone gel+ oral dydrogesterone | 58(61.70 %) | 44(47.83 %) | 0.077 |
| Other | | 12 (11.32%) | 17 (15.60 %) | 0.426 |
| Endometrial thickness before frozen embryo transfer (mm) | | 9.61 ± 1.54 | 9.60 ± 1.59 | 0.961 |
| Cleavage-stage embryo transfer | | 10 (9.43 %) | 8 (7.34 %) | 0.629 |
| Blastocyst transfer | | 96 (90.57%) | 101 (92.66 %) | 0.629 |
| Mean no. of embryos transferred | | 1.13 ± 0.33 | 1.06 ± 0.25 | 0.142 |
| 1 embryo | | 93 (87.74 %) | 102 (93.58 %) | 0.163 |
| 2 embryos | | 13 (12.26 %) | 7 (6.42 %) | 0.163 |

Values are presented as the mean ± SD or n (%). OCP, Oral contraceptive pill; FET, frozen/warmed embryo transfer; HRT, hormone replacement therapy; LPS, luteal-phase support
